# Is tumor shape associated with molecular diagnosis, extent of resection, or postoperative focal deficits in diffuse low-grade gliomas?

**DOI:** 10.1093/noajnl/vdaf138

**Published:** 2025-08-19

**Authors:** Claes Johnstad, Ingerid Reinertsen, Alba Corell, Erik Thurin, Tora Dunås, Margret Jensdottir, Jiri Bartek, Klas Holmgren, Rickard L Sjöberg, Francesco Latini, Maria Zetterling, Rupavathana Mahesparan, Peter Milos, Björn Sjögren, Henrietta Nittby Redebrandt, Gregor Tomasevic, Lars Kjelsberg Pedersen, Asgeir S Jakola, Ole Solheim

**Affiliations:** Department of Neuromedicine and Movement Science, Faculty of Medicine and Health Sciences, Norwegian University of Science and Technology, Trondheim, Norway; Department of Circulation and Medical Imaging, Faculty of Medicine and Health Sciences, Norwegian University of Science and Technology, Trondheim, Norway; Department of Health Research, SINTEF Digital, Trondheim, Norway; Department of Neurosurgery, Sahlgrenska University Hospital, Gothenburg, Sweden; Department of Clinical Neuroscience, Institute of Neuroscience and Physiology, Sahlgrenska Academy, University of Gothenburg, Gothenburg, Sweden; Department of Radiology, Sahlgrenska University Hospital, Gothenburg, Sweden; Department of Clinical Neuroscience, Institute of Neuroscience and Physiology, Sahlgrenska Academy, University of Gothenburg, Gothenburg, Sweden; Department of Clinical Neuroscience, Karolinska Institutet, Stockholm, Sweden; Department of Neurosurgery, Karolinska University Hospital, Stockholm, Sweden; Department of Neurosurgery, Rigshospitalet, Copenhagen, Denmark; Department of Clinical Neuroscience, Karolinska Institutet, Stockholm, Sweden; Department of Neurosurgery, Karolinska University Hospital, Stockholm, Sweden; Department of Clinical Science, Umeå University, Umeå, Sweden; Department of Clinical Science, Umeå University, Umeå, Sweden; Department of Medical Sciences, Section of Neurosurgery, Uppsala University Hospital, Uppsala, Sweden; Department of Medical Sciences, Section of Neurosurgery, Uppsala University Hospital, Uppsala, Sweden; Department of clinical medicine, University of Bergen, Norway; Department of Neurosurgery, Haukeland University Hospital, Bergen, Norway; Department of Neurosurgery, Skåne University Hospital, Lund, Sweden; Department of Neurosurgery, Linköping University Hospital, Linköping, Sweden; Department of Biomedical and Clinical Sciences, Linköping University Hospital, Linköping, Sweden; Department of Neurosurgery, Skåne University Hospital, Lund, Sweden; Department of Neurosurgery, Linköping University Hospital, Linköping, Sweden; Department of Biomedical and Clinical Sciences, Linköping University Hospital, Linköping, Sweden; Department of Neurosurgery, University Hospital of North Norway, Tromsø, Norway; Department of Neurosurgery, University Hospital of North Norway, Tromsø, Norway; Department of Neurosurgery, University Hospital of North Norway, Tromsø, Norway; Department of Neurosurgery, St. Olav’s Hospital, Trondheim University Hospital, Trondheim, Norway; Department of Neurosurgery, Sahlgrenska University Hospital, Gothenburg, Sweden; Department of Neurosurgery, St. Olav’s Hospital, Trondheim University Hospital, Trondheim, Norway; Department of Neurosurgery, Sahlgrenska University Hospital, Gothenburg, Sweden; Department of Neuromedicine and Movement Science, Faculty of Medicine and Health Sciences, Norwegian University of Science and Technology, Trondheim, Norway

**Keywords:** LGG, sphericity index, tumor surface area, tumor shape, tumor size

## Abstract

**Background:**

This study aimed to explore the potential association between tumor shape, 1p/19q codeletion, EOR, and new postoperative focal deficits in patients with diffuse low-grade glioma.

**Methods:**

We analyzed data from 225 WHO grade 2 glioma surgeries performed in nine centers in Norway and Sweden. The tumor measurements were based on automatic segmentations of preoperative T2/FLAIR MRI scans by Raidionics. Contact surface area (CSA) was defined as the area between the tumor and brain parenchyma and was estimated by subtracting the surface area covered by the dura from the total surface area. The sphericity index (SI) was defined as the quotient of the tumor surface area and the surface area of a sphere with equal volume. Focal deficits were defined as any new motor, language, or visual deficits postoperatively.

**Results:**

The univariable analyses showed that a larger CSA was associated with higher age (*P* = .02), lower EOR (*P* < .0001), and more focal deficits (*P* = .005) but not with 1p/19q codeletion (*P* = .54). A higher SI was associated with higher age (*P* = .02) and lower EOR (*P* < .0001) but not with focal deficits (*P* = .08) or 1p/19q codeletion (*P* = .90). The multivariable linear regression model supported the univariable associations between EOR and CSA (*P* = .0003) and SI (*P* = .0013), respectively. Contrarily, the logistic regression model showed that focal deficits were associated with SI (*P* = .014) but not with CSA (*P* = .056)

**Conclusion:**

The tumor shape appears to be independently associated with EOR and new focal deficits but not with molecular diagnosis in patients with low-grade glioma.

**Key Points**

- Tumor shape was significantly associated with the extent of resection in LGG.
- We found no association between the extent of resection and tumor volume.

**Importance of the Study**

This is the first study to assess the prognostic and diagnostic value of the contact surface area and sphericity index of low-grade gliomas. Our data show the importance of preoperative tumor shape and how well it correlates with the extent of surgical resection. Future studies on radiomics or preoperative assessment of low-grade gliomas should consider incorporating the predictive value of tumor shape and surface area.

Diffuse low-grade gliomas (LGG) in adults can be classified into two histopathological subtypes, astrocytoma and oligodendroglioma, based on the respective absence and presence of 1p/19q codeletion.^1^ While astrocytoma is associated with a worse prognosis than oligodendroglioma, with a median survival of 13 vs. 22 years,^2^ other major prognostic factors are tumor volume and extent of surgical resection (EOR). EOR above 75% is associated with longer survival with a dose response for more extensive resections, particularly for astrocytomas.^2,3^ While there is still no accurate method for distinguishing astrocytoma from oligodendroglioma in preoperative images, studies have linked 1p/19q status to fractional anisotropy in diffusion tensor imaging (DTI),^4^ T2-fluid-attenuated inversion recovery (FLAIR) mismatch,^5–7^ and signs of calcification on magnetic resonance imaging (MRI).^6,8,9^ Further, surgical risk and resectability are linked to tumor location and involvement of so-called functional brain regions.^10,11^ However, preoperatively, the major prognostic factors in patients with diffuse low-grade glioma, i.e. the molecular diagnosis and obtained extent of resection, are still unknown, which may hamper estimation of risks and benefits associated with the chosen surgical strategy.

Tumor shape has previously been suggested as a potential prognostic factor in glioblastoma patients.^12–14^ In a recent study, we assessed tumor shape in patients with glioblastoma and found that contact surface area (CSA) and sphericity index (SI) were independent prognostic factors for survival and linked to MGMT (O_6_-methylguanine-DNA methyltransferase) methylation status and EOR.^15^ We hypothesized that tumor shape could be of clinical importance in patients with LGG as well, with extent of resection (EOR) as a possible mediator. Complex shape could potentially be associated with the molecular diagnosis, affect the EOR and/or neurological outcomes, and consequently have clinical and prognostic importance. We hypothesized that the dura mater acts as a barrier to further tumor growth, and therefore, we have investigated the CSA, meaning the tumor surface area not covered by the dura, rather than the total surface area. Additionally, we have studied SI, which represents tumor shape complexity alone, without the influence of size.

The purpose of this study was to assess whether tumor shape, represented by CSA or SI, from preoperative MRI could have clinical importance by predicting molecular diagnosis and surgical outcome in terms of EOR and acquired focal deficits, in patients with World Health Organization (WHO) grade 2 gliomas.

## Methods

### Data

This project was a population-based, retrospective cohort study using the LGG STAR data, a dataset of 515 LGG patients treated in Norway or Sweden in 9 different centers from 2012 through 2017.^16^ All 243 patients with confirmed IDH mutations were included in the present study. The tumors were subclassified into oligodendrogliomas or astrocytomas based on available 1p/19q codeletion status (available for 180 patients). Patients who underwent biopsy only were not included in the analyses of EOR or acquired focal deficits. Eloquence was defined as tumor location in eloquent cortex as defined by Chang et al./UCSF, including the sensorimotor strip, dominant hemisphere perisylvian language areas, the basal ganglia, internal capsule, the thalamus, and the calcarine visual cortex.^17^

### Tumor Measurements

**Figure 1.**
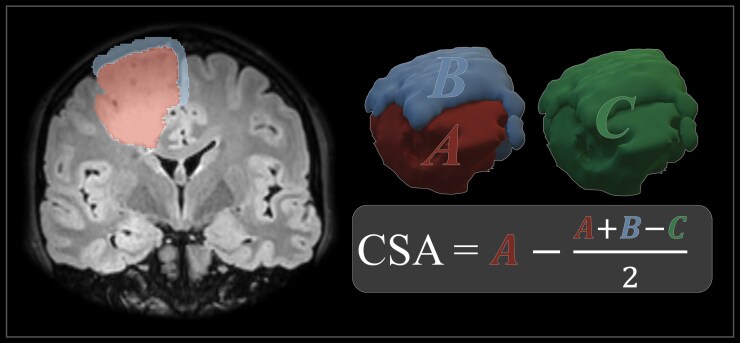
Illustration of the calculation of CSA. This example shows the segmentation of a tumor (A); the dura mater in contact with the tumor (B); and the two segmentations combined (C). The formula shows the calculation of CSA by subtraction of the surface area between the tumor and dura mater from the total tumor surface area. The letters A, B, and C in the formula represent the surface areas of their respective segmentations. In a subcortical tumor, B would be 0, and C would be equal to A, and thus, the CSA would be equal to A. CSA: contact surface area.

The volumes, SI, and CSA of the tumors were estimated using automatic segmentations of preoperative T2/FLAIR scans. The segmentations were performed by Raidionics, a deep learning-based software developed by our research group,^18^ and manually verified or corrected by a trained medical student. CSA was defined as the surface area between the tumor and adjacent brain parenchyma on MRI, excluding surface area covered by the dura. To find the CSA of the tumor, a trained medical student manually subtracted the surface area in contact with the dura mater from the total surface area, using 3D Slicer, version 5.6.1.^19,20^Figure 1 shows an example of a segmentation and the formula for calculating CSA. SI was defined as the quotient of the surface area of the tumor and the surface area of a sphere with equal volume as the tumor. Thus, a higher SI means a less spherical shape.

### Outcome Variables

The main outcome variables in this study were EOR and focal deficits. EOR was analyzed as a continuous variable defined as the percentwise decrease in measurable tumor volume from preoperative to postoperative MRI, using T2/FLAIR sequences. It was only categorized into tertiles of subtotal resection and gross total resection for illustrative purposes in Figure 3. Focal acquired deficits were defined as any clinician-reported new or worsened language, motor, and/or visual deficits registered at discharge.

### Statistical Analyses

All statistical analyses were conducted in RStudio, version 2023.06.0 + 421.^21^ Distribution of the data was determined non-normal by density curves, Q-Q plots, and Shapiro-Wilk test, and the relevant non-parametric methods were utilized to calculate potential associations between the defined shape variables and endpoints. P values of less than 0.05 were considered statistically significant. A logistic regression model and a Huber weights robust linear regression model were used to assess the significant associations in the univariable analyses with adjustments for important covariates. A logistic regression model was used for the binary outcome variable focal deficits, whereas a linear regression model that is less sensitive to outliers in the data was used for the continuous outcome variable EOR. The covariates were selected based on significant associations in the univariable analyses.

### Ethics

The study was approved by the regional committee of Western Sweden (EPN reference 705/17) and the Regional Committee for Medical and Health Research Ethics in Central Norway (REC reference 2017/1780). The need for informed consent was waived by the committees.

## Results

**Figure 2.**
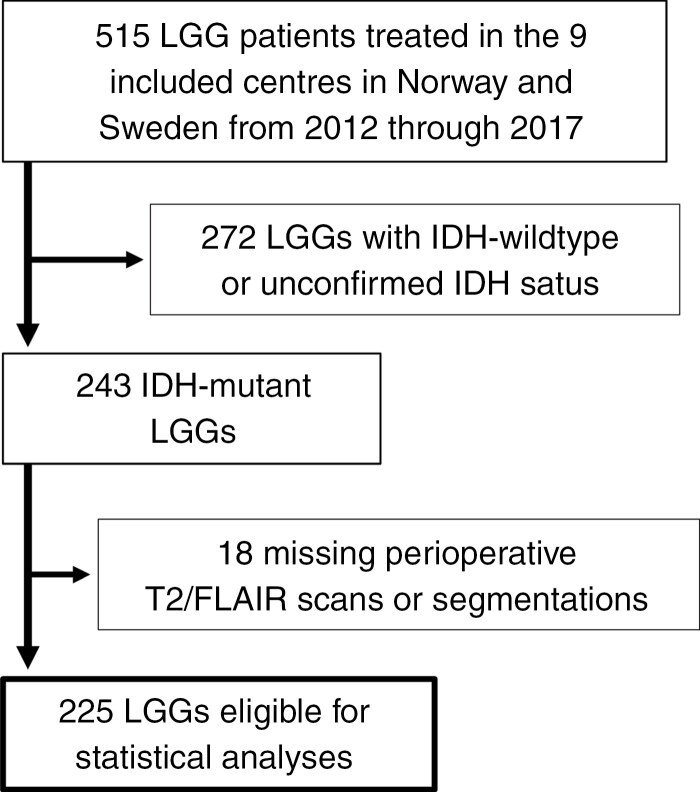
Flowchart of the inclusion process. LGG: low-grade glioma; IDH: isocitrate dehydrogenase; FLAIR: fluid-attenuated inversion recovery.

Figure 2 shows a flowchart of the inclusion process. Out of the total number of 515 LGG patients treated in the relevant centers, 225 patients remained eligible for this study. Baseline characteristics of the included patients are presented in Table 1.

**Table 1.** Data Characteristics

Continuous variables	Median (1^st^ quartile—3^rd^ quartile)
Age	39 (31-51)
Tumor volume (cm^3^)	32.0 (12.8-70.3)
CSA (cm^2^)	60.9 (32.4-119.7)
SI	1.57 (1.36-1.93)
EOR (N = 198)	0.86 (0.65-1.0)
Categorical variables	N (%)
**Sex**	
Male	135 (60%)
Female	90 (40%)
**Tumor location**	
Frontal	132 (59%)
Temporal	46 (20%)
Parietal	22 (10%)
Occipital	4 (2%)
Insula	19 (8%)
Central/deep/basal ganglia/thalamus	2 (1%)
Eloquent location*	
Yes	136 (61%)
No	88 (39%)
**Molecular diagnosis**	
Astrocytoma	83 (37%)
Oligodendroglioma	97 (43%)
Not analyzed	45 (20%)
**Surgery**	
Resection	201 (89%)
Biopsy only	24 (11%)
**Focal deficits**	
Yes	31 (15%)
No	170 (85%)
**EOR**	
GTR	51 (26%)
STR	147 (74%)

CSA: contact surface area; SI: sphericity index; EOR: extent of resection; GTR: gross total resection; STR: subtotal resection. *Defined as location in presumed eloquent cortex as defined by Chang/UCSF^17^.

**Figure 3.**
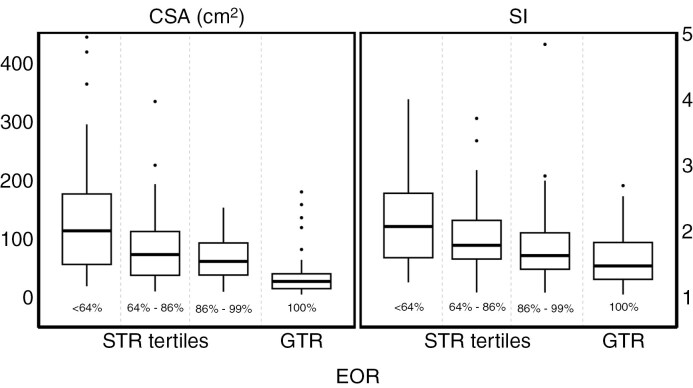
Boxplot showing CSA and SI among different EOR groups. STR is grouped by tertiles with *n* = 49 for each box, whereas GTR is represented by its own box with *n* = 51. EOR range for the STR tertiles is displayed below the respective boxes. The EOR was categorized in this plot for illustrative purposes, but it was otherwise analyzed as a continuous variable. EOR: extent of resection; CSA: contact surface area; SI: sphericity index; STR: subtotal resection; GTR: gross total resection.

Table 2 shows a summary of the univariable statistical analyses. In the univariable analyses, CSA was significantly associated with age, EOR, and acquired focal neurological deficits at discharge. SI was significantly associated with age and EOR, but not with focal deficits. No other statistically significant associations were observed. Figure 3 illustrates the unadjusted association between EOR categorized into four groups and CSA and SI, respectively. In addition to gross total resection being strongly associated with a lower CSA or SI, there were also considerable differences between those who underwent moderate versus extensive subtotal resection.

**Table 2.** Univariable Analyses

	CSA		SI	
Variable	Test statistic (variable vs. CSA)	*P*-value	Test statistic (variable vs. SI)	*P*-value
Spearman Correlation Test				
	Correlation coefficient		Correlation coefficient	
Age	0.15	**0.02**	0.16	**0.02**
EOR	−0.49	**<0.0001**	−0.38	**<0.0001**
Mann-Whitney U test				
	Median CSA (Q_1_-Q_3_)		Median SI (Q_1_-Q_3_)	
** Sex**		0.46		0.77
Male (n = 135)	57 (33-139)		1.59 (1.36-1.98)	
Female (n = 90)	63 (30-105)		1.52 (1.37-1.91)	
** Histological subtype**		0.54		0.90
Astrocytoma (n = 83)	69 (31-142)		1.62 (1.36-2.03)	
Oligodendroglioma (n = 97)	61 (39-105)		1.57 (1.40-1.99)	
** Acquired focal deficits**		**0.04**		0.08
Yes (n = 31)	77 (36-130)		1.42 (1.31-1.72)	
No (n = 170)	54 (29-103)		1.57 (1.36-1.93)	
Kruskal-Wallis test				
	Median CSA (Q_1_-Q_3_)		Median SI (Q_1_-Q_3_)	
** Location**		0.11		0.06
Frontal (n = 132)	51 (29-112)		1.49 (1.35-1.85)	
Temporal (n = 46)	70 (41-146)		1.66 (1.48-2.24)	
Parietal (n = 22)	53 (34-108)		1.69 (1.31-1.87)	
Occipital (n = 4)	43 (26-76)		1.45 (1.36-1.55)	
Insula (n = 19)	85 (70-139)		1.72 (1.52-2.14)	
Central/Deep/Basal ganglia/ Thalamus (n = 2)	122 (72-173)		1.64 (1.46-1.81)	

CSA: contact surface area; SI: sphericity index; EOR: extent of resection; Q_1_: First quartile; Q_3_: Third quartile.

Two multivariable regression analyses were conducted based on the results of the univariable analyses and are presented in Table 3. The Huber weights robust linear regression models showed a significant association between EOR and CSA (*P* = .0003) as well as SI (*P* = .0013), when adjusting for preoperative tumor volume, age, eloquence, and lobar location. The logistic regression models showed a significant association between focal deficits and SI (*P* = .014) but not CSA (*P* = .056) when adjusting for preoperative tumor volume, age, and eloquence.

**Table 3.** Multivariable Analyses

Huber Weights Robust Linear Regression Models on EOR		
Predictor variables	*Coefficient (95% confidence interval)*	*P-value*
**CSA Model**		
Age	−0.0018 (−0.0037, −0.0001)	**.061**
Preoperative volume	0.0004 (−0.0006, 0.0013)	.44
CSA	−0.0012 (−0.0019, −0.0006)	**.0003**
Eloquent location	−0.10 (−0.15, −0.04)	**.0006**
Lobar location		
Frontal	0.17 (−0.09, 0.42)	.22
Insula	0.09 (−0.17, 0.36)	.49
Occipital	0.17 (−0.15, 0.49)	.32
Parietal	0.18 (−0.09, 0.44)	.18
Temporal	0.13 (−0.13, 0.38)	.39
**SI Model**		
Age	−0.0020 (−0.0039, −0.0001)	**.036**
Preoperative volume	−0.0008 (−0.0014, −0.0003)	**.0028**
SI	−0.097 (−0.155, −0.039)	**.0013**
Eloquent location	−0.11 (−0.17, −0.06)	**.0001**
Lobar location		
Frontal	0.17 (−0.08, 0.43)	.19
Insula	0.095 (−0.172, 0.363)	.49
Occipital	0.16 (−0.17, 0.48)	.35
Parietal	0.18 (−0.08, 0.45)	.18
Temporal	0.13 (−0.12, 0.39)	.31

Logistic Regression Models on Focal Deficits		
Predictor variables	*Odds ratio (95% confidence interval)*	P-value
**CSA Model**		
Age	1.027 (0.997, 1.058)	.079
Preoperative volume	1.021 (1.008, 1.034)	**.0015**
CSA	0.991 (0.980, 0.999)	.056
Eloquent location	1.328 (0.574, 3.213)	.51
**SI Model**		
Age	1.029 (0.999, 1.061)	.060
Preoperative volume	1.013 (1.005, 1.021)	**.0011**
SI	0.258 (0.079, 0.702)	**.014**
Eloquent location	1.283 (0.556, 3.084)	.56

CSA: contact surface area; SI: sphericity index: EOR: extent of resection.

## Discussion

In this retrospective, population-based multicenter study, we explored the potential prognostic value of tumor shape, represented by CSA or SI, in LGG patients. The CSA was independently associated with the EOR regardless of tumor volume. However, the CSA predicted neither molecular diagnosis nor postoperative focal deficits in our analyses. SI was associated with EOR and new focal deficits, but not with molecular diagnosis.

While the prognostic importance of tumor size and location has been extensively studied, the potential importance of tumor shape has, to our knowledge, not been studied for diffuse LGG. Similar to our findings in glioblastomas,^15^ the current study showed that surgeons typically leave more of the complexly shaped than the spherically shaped tumors.

Although higher CSA does not necessarily mean a less regular shape, the adjusted analysis showed that the finding was not merely a factor of preoperative tumor volume, age, and tumor location. Additionally, our analysis of SI supported the association between tumor shape complexity and EOR. It may be explained by the difficulties of grossly resecting an irregularly shaped tumor without damaging healthy brain tissue or by the issues of distinguishing glioma tissue from healthy parenchyma around a less spherical tumor. Although we see a link between tumor shape complexity and EOR in our data, there is not necessarily a direct cause-and-effect relationship. The observed association could be mediated by factors such as tumor infiltration pattern or involvement of white matter tracts, which again leads to a more conservative approach by the surgeon. Nevertheless, LGGs with a large CSA or SI represent a risk for lower EOR, which has been repeatedly linked with lower survival rates and poorer functional outcomes.^2,22–25^ These results suggest that a complex tumor shape could be a negative prognostic sign in patients with LGG.

The other observed response variable, focal deficits, was a composite endpoint comprising language, motor, and visual deficits. Acquired focal neurological deficits at discharge were associated with larger CSA, but not with SI, in the univariable analyses. When adjusting for age, preoperative tumor volume, and eloquence, only SI was significantly associated with focal deficits. Although a lack of power may in part explain the insignificant predictive value of CSA in the logistic regression model, it could also be a consequence of adjusting for tumor eloquence and volume. Presumed eloquent tumor location was associated with increased risk of postoperative neurological deficits,^26^ whereas tumor volume was significantly associated with focal deficits in our data, although contradicting former research.^26,27^ Another factor that could potentially mask the association between CSA and acquired focal deficits is the surgeon’s approach to a larger, more deeply located tumor perhaps being more conservative, as evidenced by the observed lower EOR and the presumably lesser damage to adjacent parenchyma. Although this could also be a result of function-based resection, only 35% of the patients underwent awake surgery or asleep surgery with functional brain mapping. However, also in image-guided or anatomical landmark-guided surgery, the more complexly shaped tumors would be more difficult to fully resect, which could explain why larger CSA was associated with lower EOR but not focal deficits in our data. SI, on the other hand, was significantly associated with focal deficits in the multivariable model. This could suggest that the tumor shape complexity itself—without the influence of tumor volume and location, which must be considered in CSA—predicts the development of new focal deficits. However, this outcome variable was obtained early postoperatively and may only express transient deficits with less clinical importance.

The SI is a quite simple measurement that represents the shape complexity of the tumor only. The CSA, however, is a complex measurement and comprises the shape, size, and location of an LGG. An irregular tumor shape, larger tumor volume, and deeper tumor location will all contribute to a larger CSA. When adjusting for volume and location, CSA does not independently seem to predict focal deficits but is independently associated with EOR. The mechanism behind this correlation is not clear but could be mediated by the surgeon’s approach or confounded by the location of the tumor beyond the extent of the location-wise adjustments performed in the current study. The analyses of SI support our initial hypothesis that the shape complexity alone, regardless of tumor volume and location, is a significant predictor of EOR and may also predict postoperatively acquired focal deficits.

Despite numerous machine learning and radiomics studies on the subject, there are, to our knowledge, no clinically applicable, robust methods for non-invasive prediction of 1p/19q co-deletion. Based on previously observed associations between tumor shape and molecular subtype,^28^ we hypothesized that CSA or SI could help distinguish astrocytomas from oligodendrogliomas on preoperative MRI. However, in line with a previous radiogenomics study on a different shape measurement,^29^ there was no association between 1p/19q status and tumor shape in our data.

The clinical rationale for this study was to assess new potential radiological features that could be prognostically important in management of LGG. Tumor diameters have long been the gold standard for measuring gliomas and are linked to survival,^17^ but it oversimplifies the tumor size, particularly for non-spherical tumors. Tumor volume is more accurate and is linked to survival,^2^ but there is no compelling association between this measurement and EOR in LGGs. Surgical operability and risk are linked to tumor location and eloquence, but there may be other important radiological factors. Thus, we hypothesized that tumor shape complexity could be a better prognostic feature on preoperative MRI for surgical planning in LGG patients, and these results support that hypothesis. MRI is an important tool in preoperative planning, and being able to assess the molecular diagnosis, prognosis, and resectability of the tumor noninvasively would be very helpful when weighing risks against benefits of surgery. In this study, we were not able to predict the 1p/19 codeletion status of LGGs, but we found a valuable radiological phenotype for prediction of resectability and focal deficits. While diffuse gliomas may appear as a well-defined mass, easily distinguishable from surrounding tissue on MRI, tumor cells infiltrate diffusely beyond the radiological margins of the tumor.^30,31^ Thus, the radiological extent of the tumor is not a perfect representation of its infiltration, but it is still how EOR and tumor progression are classified. It could be possible that more complexly shaped gliomas harbor a larger unmeasured infiltration zone, and to what extent this is linked to survival is not yet known.

This is, as far as we know, the first study to investigate the prognostic importance of tumor shape in LGGs. The greatest strengths of this study were the population-based sample from multiple centers and verified tumor segmentations improving both external and internal validity of the results. Limitations include the retrospective approach impeding control over data collection and a relatively small cohort with focal deficits and lobar locations, thus limiting the power of the relevant analyses. Additionally, because IDH status has been increasingly more relevant in later years, a lot of patients treated for LGG during the first years of the data collection were excluded due to missing IDH status, which could introduce a selection bias.

## Conclusion

The tumor shape complexity on preoperative MRI is significantly associated with the extent of surgical resection and may also be linked with new postoperative focal deficits in patients with WHO grade 2 gliomas. However, it does not predict histopathological subtype.

## Data Availability

The datasets presented in this article are not readily available because of restricted access by the General Data Protection Regulation (GDPR). Requests to access the datasets should be directed to Claes.Johnstad@ntnu.no.
